# Rare loss of function mutations in *N*-methyl-d-aspartate glutamate receptors and their contributions to schizophrenia susceptibility

**DOI:** 10.1038/s41398-017-0061-y

**Published:** 2018-01-10

**Authors:** Yanjie Yu, Yingni Lin, Yuto Takasaki, Chenyao Wang, Hiroki Kimura, Jingrui Xing, Kanako Ishizuka, Miho Toyama, Itaru Kushima, Daisuke Mori, Yuko Arioka, Yota Uno, Tomoko Shiino, Yukako Nakamura, Takashi Okada, Mako Morikawa, Masashi Ikeda, Nakao Iwata, Yuko Okahisa, Manabu Takaki, Shinji Sakamoto, Toshiyuki Someya, Jun Egawa, Masahide Usami, Masaki Kodaira, Akira Yoshimi, Tomoko Oya-Ito, Branko Aleksic, Kinji Ohno, Norio Ozaki

**Affiliations:** 1 Department of Psychiatry, Nagoya University Graduate School of Medicine, Nagoya, Japan; 2 Division of Neurogenetics, Center for Neurological Diseases and Cancer, Nagoya University Graduate School of Medicine, Nagoya, Japan; 3 Shengjing Hospital of China Medical University, Shenyang, China; 4 Institute for Advanced Research, Nagoya University, Nagoya, Japan; 5 Brain and Mind Research Center, Nagoya University, Nagoya, Japan; 6 Center for Advanced Medicine and Clinical Research, Nagoya University Hospital, Nagoya, Japan; 7 Laboratory for Psychiatric and Molecular Neuroscience, McLean Hospital, Belmont, MA 02478 USA; 8 Department of Psychiatry, Fujita Health University School of Medicine, Toyoake, Japan; 9 Department of Neuropsychiatry Okayama University Graduate School of Medicine, Dentistry and Pharmaceutical Sciences, Okayama, Japan; 10 Department of Psychiatry, Niigata University Graduate School of Medical and Dental Sciences, Niigata, Japan; 11 Department of Child and Adolescent Psychiatry Kohnodai Hospital, National Center for Global Health and Medicine, Tokyo, Japan; 12 Division of Clinical Sciences and Neuropsychopharmacology, Faculty and Graduate School of Pharmacy, Meijo University, Nagoya, Japan; 13 Department of Nutrition, Shubun University, Ichinomiya, Japan

## Abstract

In schizophrenia (SCZ) and autism spectrum disorder (ASD), the dysregulation of glutamate transmission through *N*-methyl-d-aspartate receptors (NMDARs) has been implicated as a potential etiological mechanism. Previous studies have accumulated evidence supporting NMDAR-encoding genes' role in etiology of SCZ and ASD. We performed a screening study for exonic regions of *GRIN1*, *GRIN2A*, *GRIN2C*, *GRIN2D*, *GRIN3A*, and *GRIN3B*, which encode NMDAR subunits, in 562 participates (370 SCZ and 192 ASD). Forty rare variants were identified including 38 missense, 1 frameshift mutation in *GRIN2C* and 1 splice site mutation in *GRIN2D*. We conducted in silico analysis for all variants and detected seven missense variants with deleterious prediction. De novo analysis was conducted if pedigree samples were available. The splice site mutation in *GRIN2D* is predicted to result in intron retention by minigene assay. Furthermore, the frameshift mutation in *GRIN2C* and splice site mutation in *GRIN2D* were genotyped in an independent sample set comprising 1877 SCZ cases, 382 ASD cases, and 2040 controls. Both of them were revealed to be singleton. Our study gives evidence in support of the view that ultra-rare variants with loss of function (frameshift, nonsense or splice site) in NMDARs genes may contribute to possible risk of SCZ.

## Introduction

Schizophrenia (SCZ)and autism spectrum disorder (ASD) both have been implied to a high heritability and a strong genetic basis^1,2^. SCZ is a common, serious mental disorder which affects nearly 1% people of the world^3^. Twin studies estimated its heritability to be up to 80%^4^. ASD is a range of heterogeneous neurodevelopmental conditions which has early-onset deficiency in social communication and interactions, and also behavioral functioning^5^. The etiology of ASD is strongly impacted by genetics, with heritability estimates of 56–95%^6^.

Glutamate is one of the most important excitatory neurotransmitter in synapse systems and 40% of all synapses^7^ are exploiting it. Glutamate is involved in many central nervous system processes and basic neuronal functions^8^. Thus, abnormal glutamatergic neurotransmission could be a point of convergence to describe the neurocognitive deficits and feature of symptoms presented in nervous system diseases^8–10^. *N*-methyl-d-aspartate receptors (NMDARs) indicated to be one of the most common glutamate receptors. The pathology of anti-NMDAR encephalitis implies that abnormalities in glutamatergic signaling can result in cognitive impairment, mood changes, and impairment of behavior, which are the symptoms often observed in SCZ/ASD patients^11,12^. To date, NMDAR subunits have three main family members that have been identified: NR1, NR2, and NR3. Those subunits are encoded by *GRIN1*, *GRIN2A*, *GRIN2B*, *GRIN2C*, *GRIN2D*, *GRIN3A*, and *GRIN3B* ^13,14^, respectively.

Resequencing studies suggested that *GRIN2A* might be a candidate gene for autism and SCZ^15–17^ A genomic data analysis study suggests *GRIN2C* as another SCZ candidate gene^18^. An exome sequencing study which sequenced all *GRIN* genes in SCZ and ASD cases, detected de novo variants in *GRIN2B* and *GRIN2A* and loss-of-function (LoF) variants in *GRIN2C*, *GRIN3A*, and *GRIN3B* ^19^. Novel de novo microduplications in 19q13, where *GRIN2D* resides in, were identified in ASD^20^. In addition, a study that identified significant association between *GRIN3B* and mismatch negativity (MMN) also deserves attention, as decreasing MMN was suggested to be correlated with the pathogenesis of SCZ^21^. Furthermore, in mouse studies, *GRIN2A*-null mice exhibit some SCZ-like symptoms^22^. Studies with *GRIN2C* knockout mice pointed out NR2C receptors might play a potential role in associative and executive learning^23^. Further mice studies showed that NR2D subunit incorporates into the NMDARs that mediate excitatory synaptic transmission onto interneurons and influence interneuron function and signal processing^24^. Altogether, these findings suggested the importance of *GRIN* genes in the pathogenesis of SCZ and ASD.

Recently, there have been accumulating evidence supporting a role of rare variants in mental disorders causation^25–27^, especially rare LoF (nonsense, splice site or frameshift) variants^28–30^. While frameshift and nonsense mutations are clearly to be LoF, the effect of splice site mutation remains to be defined. To our knowledge, till now, previous papers have not put their attention on *GRIN gene*s splice site mutation in cases of neuropsychiatric disorders. In previous work^31^, we sequenced *GRIN2B* in SCZ and ASD, identifying five rare missense mutations. In present study, we performed a mutation screening study for the exonic regions of *GRIN1*,* GRIN2A*, *GRIN2C*,* GRIN2D*, *GRIN3A*, and *GRIN3B*. We assessed the functional impact of splice site mutation by minigene assay, and performed genotyping for LoF mutations in a large sample set.

## Methods

### Samples

In this study, two independent sample groups were designed (Table 1). The first one, which included 370 SCZ and 192 ASD patients, resequencing for mutation discovery. The second one, with 1877 SCZ cases, 382 ASD cases, and 2040 healthy controls, was used for genotyping of selected mutations identified in the first step. All participates in our study are ethnically Japanese, live on the mainland of Japan. The Hospital of Nagoya University and its co-institutes (Toyama University, Niigata University, Fujita Health University) and co-hospital (Kohnodai Hospital) recruited all the participates. Patients included in the study were diagnosed according to Diagnostic and Statistical Manual of Mental Disorders, Fifth Edition criteria for SCZ or ASD. Controls were recruited from ordinary people and were evaluated with an unstructured interview to ensure that they never suffer from psychiatric disorders, both personal and family history. We explained our study to all participants both verbally and in writing. In addition, if individuals had no capacity to do this alone, their parents or other family members were required to complete written informed consent. The study was supported by Ethics Committees of the Nagoya University Graduate School of Medicine and co-institutes and co-hospital.

**Table 1:** Profiles of samples in the small set for resequencing and the large set for association analysis

	Resequencing		Association analysis		
	SCZ	ASD	SCZ	ASD	Controls
Total	370	192	1877	382	2040
Male	196 (52.97%)	149 (77.60%)	1027 (54.71%)	297 (77.75%)	998 (48.92%)
Female	174 (47.03%)	43 (22.40%)	850 (45.29%)	85 (22.25%)	1042 (51.08%)
Mean age (years)	49.73 ± 14.75	16.34 ± 8.36	46.87 ± 15.35	19.61 ± 10.71	46.89 ± 14.61

*SCZ* schizophrenia; *ASD* autism spectrum disorders,

age at recruitment

### Sequencing and data collection

We extracted genomic DNA from whole peripheral blood or saliva by a standard protocol. For covering coding regions of *GRIN1*, *GRIN2A*, *GRIN2C*, *GRIN2D*, *GRIN3A*, and *GRIN3B* (human reference sequence NCBI (build 37)), we designed custom amplification primers by FastPCR (PrimerDigital Ltd, Helsinki, Finland) and NCBI Primer-BLAST. The Ion Library Equalizer Kit Adapters and Ion AmpliSeq Library Kits 2.0 (Thermo Fisher Scientific, Foster City, CA, USA) were used for amplification and equalization. Then Ion Xpress Barcode was used to collect the amplified sequence. We used Ion Torrent PGM™ (Thermo Fisher Scientific) to sequence the products by next-generation sequencing technology. Then we performed an analysis for the resulting data using Ingenuity Variant Analysis (Qiagen Ltd, Hilden, Germany).

### Filter conditions and in silico analysis

Rare (minor allele frequency < 1%), nonsynonymous variants, which was located on the functional domain, under the Human Protein Reference Database (http://www.hprd.org), EMBL-EBI (https://www.ebi.ac.uk/), and the existing literatures (Table 2), were selected from the original data for further analysis. These filtered variants were then sequenced for confirming their reliability by Sanger method in a 3130XL Genetic Analyzer (Applied Biosystems, Foster City, CA, USA).

**Table 2:** Details of discovered rare mutations and in silico analyses

a																	
Chromosome	Position	Reference allele	Sample allele	Gene symbol	Phenotype	Protein variant	dbSNP ID	1000 Genomes frequency	NHLBI ESP frequency	HGVD frequency	iJGVD frequency	SIFT function prediction	PolyPhen-2 function prediction	MUTATIONTASTER function prediction	PRIMATE phcons	Primate phylop	Pedigree analysis
9	14,0052,907	G	A	*GRIN1*	SCZ	p.A349T; p.A370T	Rs148008303	0.0002	0.00006			Damaging	Possibly Damaging	disease causing	0.993	0.557	
16	9,858,015	T	C	*GRIN2A*	SCZ	p.H1129R			0.000016			Tolerated	Benign	disease causing	0.074	0.525	
16	9,858,403	C	T	*GRIN2A*	ASD	p.V1000M						Tolerated	Possibly Damaging	disease causing	0.959	0.651	inherited
16	9,858,511	G	C	*GRIN2A*	SCZ	p.Q964E			0.000032		0.0005	Tolerated	Benign	disease causing	0.983	0.651	
16	9,858,751	C	T	*GRIN2A*	SCZ	p.D884N	Rs777684328	0.00002		0.0009	Damaging	Possibly Damaging	disease causing	0.994	0.651		
16	9,858,774	A	G	*GRIN2A*	ASD	p.I876T	Rs199784503	0.0004	0.0003		0.0023	Tolerated	Probably Damaging	disease causing	0.965	0.53	inherited
16	9,862,785	G	A	*GRIN2A*	ASD	p.L840F	Rs371352783	0.00002			Damaging	Possibly Damaging	disease causing	0.997	0.559	inherited	
16	9,927,969	T	G	*GRIN2A*	ASD	p.K590N			0.000033		0.0005	Tolerated	Benign	disease causing	0.985	0.525	
16	9,943,618	T	G	*GRIN2A*	ASD	p.K441N						Tolerated	Benign	disease causing	0.995	0.525	
16	10,032,377	G	A	*GRIN2A*	SCZ	p.A149V		0.04			0.0009	Tolerated	Probably Damaging	disease causing	0.956	0.559	
16	10,032,405	G	C	*GRIN2A*	SCZ	p.P140A			0.000008		0.0009	Tolerated	Benign	disease causing	0.932	0.559	
17	72,839,530	C	T	*GRIN2C*	ASD	p.A916T				0.0014	0.0005	Tolerated	Probably Damaging	disease causing	0.981	0.45	inherited
17	72,846,024	C	T	*GRIN2C*	ASD	p.E514K				0.0017		Tolerated	Possibly Damaging	disease causing	0.992	0.486	
17	72,846,374	G	A	*GRIN2C*	SCZ	p.R488C	Rs186790306	0.0014	0.00007			Damaging	Probably Damaging	disease causing	0.994	0.486	
17	72,846,483	C	G	*GRIN2C*	SCZ	p.K451N						Damaging	Probably Damaging	disease causing	0.722	−0.347	
17	72,846,705	G	A	*GRIN2C*	ASD	p.H439Y						Tolerated	Benign	disease causing	0.015	0.557	inherited
17	72,846,800	G	A	*GRIN2C*	SCZ	p.T407M	Rs536926397	0.0002	0.00002			Damaging	Probably Damaging	disease causing	0.318	0.55	

b																	
Chromosome	Position	Reference allele	Sample allele	Gene symbol	Phenotype	Protein cariant	dbSNP ID	1000 Genomes frequency	NHLBI ESP Frequency	HGVD Frequency	iJGVD Frequency	SIFT Function Prediction	PolyPhen-2 Function Prediction	MUTATIONTASTER Function Prediction	Primate PhCons	primate PhyloP	Pedigree analysis
17	72,850,836	GGGG		*GRIN2C*	SCZ	p.P132fs*59					NA	NA	disease causing	0.477	0.645		
17	72,850,840	G	A	*GRIN2C*	ASD	p.T131I	Rs780165386	0.000009			Damaging	Possibly Damaging	disease causing	0.401	−0.176		
17	72,851,093	G	A	*GRIN2C*	SCZ	p.R47C	Rs776102062	0.00003			Tolerated	Benign	disease causing	0.867	−0.276		
19	48,908,432	G	A	*GRIN2D*	ASD	p.G303R						Tolerated	Possibly Damaging	disease causing	0.401	0.557	inherited
19	48,908,447	C	T	*GRIN2D*	SCZ	p.R308C	Rs746751166	0.00002			Tolerated	Benign	disease causing	0.989	0.557		
19	48,917,841	G	A	*GRIN2D*	SCZ	p.S471N						Tolerated	Benign	disease causing	0.992	0.651	
19	48,918,122	C	A	*GRIN2D*	SCZ	p.P472T						Tolerated	Possibly Damaging	disease causing	0.94	0.651	
19	48,945,428	G	A	*GRIN2D*	SCZ	p.R821Q	Rs767410370	0.000008			Tolerated	Probably Damaging	disease causing	0.966	0.451		
19	48,947,106	C	T	*GRIN2D*	ASD	p.P1308L				0.0049	0.0028	deleterious	NA	disease causing	0.038	0.466	
9	104,375,732	C	A	*GRIN3A*	ASD	p.G898W					0.0005	Damaging	Probably Damaging	disease causing	0.948	0.651	
9	104,433,232	T	C	*GRIN3A*	ASD/SCZ	p.K488E	Rs189425146 0.0042	0.42	0.0009		0.0009	Tolerated	Benign	disease causing	0.985	0.53	inherited
9	104,449,017	C	T	*GRIN3A*	SCZ	p.V389I	Rs200120504	0.0002		0.0005	Tolerated	Benign	disease causing	0.99	0.559		
9	104,449,173	G	A	*GRIN3A*	ASD/SCZ	p.R337W	Rs773593066	0.00008	0.0017	0.0009	Damaging	Possibly Damaging	disease causing	0.971	0.559	inherited	
9	104,499,853	G	T	*GRIN3A*	ASD	p.R137S	Rs769491656				Tolerated	Benign	disease causing	0.988	0.651	inherited	
19	1,003,244	C	T	*GRIN3B*	SCZ	p.T181I	Rs540094501	0.0004	0.0002	0.0087	0.2924	Tolerated	Benign	disease causing	0.002	−0.397	
19	1,003,255	G	A	*GRIN3B*	SCZ	p.G185S	Rs575985258	0.001	0.0006	0.0005	0.0005	Tolerated	Benign	disease causing	0.016	−0.397	
19	1,003,721	G	A	*GRIN3B*	SCZ	p.R340Q	Rs577413695	0.0002				Tolerated	Benign	disease causing	0.652	0.459	
19	1,004,573	G	A	*GRIN3B*	SCZ	p.G358D	Rs75047944	0.0006	0.0001	0.0044	0.0089	Damaging	Probably Damaging	disease causing	0.054	−0.524	
19	1,004,998	G	A	*GRIN3B*	ASD/SCZ	p.V500M	Rs377572345	0.0001	0.0014		Damaging	Probably Damaging	disease causing	0.192	0.48		
19	1,005,098	G	T	*GRIN3B*	ASD	p.S533I	Rs200427089	0.0004	0.0002	0.0036	0.002	Damaging	Possibly Damaging	disease causing	0.933	0.48	inherited
19	1,005,209	T	A	*GRIN3B*	SCZ	p.M570K	Rs750803476	0.0001			Tolerated	Possibly Damaging	disease causing	0.863	0.393		
19	1,005,523	G	A	*GRIN3B*	SCZ	p.E675K	Rs759438437	0.000009	0.0005		Damaging	Probably Damaging	disease causing	0.026	−0.863		
19	1,008,142	A	G	*GRIN3B*	SCZ	p.Y773C				0.0009		Tolerated	Probably Damaging	disease causing	0.081	−0.897	

Genomic position is based on GRCh37/hg19

*SNV*single-nucleotide variant

*dbSNP*: dbSNP build 139 (http://www.ncbi.nlm.nih.gov/projects/SNP/); *1000 Genomes* the 1000 Genomes Project (http://www.1000genomes.org), *NHLBI* Exome Aggregation Consortium (http://exac.broadinstitute.org), *HGVD* the Human Genetic Variation Database (http://www.genome.med.kyoto-u.ac.jp/SnpDB/), *iJGVD* Integrative Japanese Genome Variation (https://ijgvd.megabank.tohoku.ac.jp/)

SIFT (http://sift.jcvi.org/), *PolyPhen-2* polymorphism phenotyping v.2 (http://genetics.bwh.harvard.edu/pph2/index.shtml). MUTATIONTASTER (http://www.mutationtaster.org/). PhastCons conservation score (http://compgen.cshl.edu/phast/phastCons-HOWTO.html): produces predictions of discrete conserved elements

We further analyzed those variants with the following methods: (1) we explored whether they were registered in the NCBI dbSNP database (build 137) (http://www.ncbi.nlm.nih.gov/SNP/), the Exome Aggregation Consortium (http://evs.gs.washington.edu/EVS/), the 1000 Genomes Project (http://www.1000genomes.org/), the Integrative Japanese Genome Variation (https://ijgvd.megabank.tohoku.ac.jp/), or the Human Genetic Variation Database (http://www.hgvd.genome.med.kyoto-u.ac.jp/index.html); (2) we looked for a possible impact of amino acid substitutions as predicted by PolyPhen-2 (http://genetics.bwh.harvard.edu/pph2/)^32^, SIFT (http://sift.jcvi.org/)^33^, and MUTATIONTASTER (http://www.mutationtaster.org/)^34^; (3) we investigated the conservation status using the PhastCons conservation score and PhyloP scores using the single-nucleotide variants scoring tool Combined Annotation Dependent Depletion^35^ (Table 2).

### Splicing in silico analysis

For the splice site mutation, for predicting splicing outcomes of mutations leading to 5′-splice site splicing defects, we performed the following in silico analysis: SD-Score^36^, human splice finder^37^, and MaxEntScan^38^ (Table 3).

**Table 3:** Details of loss-of-function mutations identified in NMDAR subunits

Position	Translation impact	Exon	Gene name	Nucleotide variant	Protein variant	Phenotype	Case sample with variant	Frequency in database				In sili*co* analysis for amino acid substitution			Splice site in sili*co* analysis		
								1000 Genomes	NHLBI	HGVD	iJGVD	PolyPhen-2	SIFT	MUTATIONTASTER	SD-Score	Human splice finder	MAXENT
17:72,850,836	Frameshift	2	*GRIN2C*	delGGGG	P132FsX192	SCZ	1/370	Not registered	Not registered	Not registered	Not registered	NA	NA	Disease causing			
19:48,917,841	Splice site	5	*GRIN2D*	G>A	S471X472	SCZ	1/370	Not registered	Not registered	Not registered	Not registered	Benign	Tolerated	Disease causing	Aberrant	Most probably affecting splicing	−1.87

*NA* not applicable; genomic position is based on GRCh37/hg19. Amino acid change based on NCBI reference sequence NP_060138.1

D-Score (http://www.med.nagoya-u.ac.jp/neurogenetics/SD_Score/sd_score.html), human splice finder (http://www.umd.be/HSF3/); MaxEntScan (http://genes.mit.edu/burgelab/maxent/Xmaxentscan_scoreseq.html)

### Construction of the plasmid containing the *GRIN2D* minigene

We constructed the c.1412G>A-*GRIN2D* minigene in the pcDNA3.1(+) vector by amplifying the 5′-end of exon 4 (starting from the second nucleotide of exon 4 to retain the normal open reading frame) to the 3′-end of exon 6 of *GRIN2D* from genomic DNA, which was extracted from the c.1412G>A-*GRIN2D* mutant sample and one healthy sample using the proofreading DNA polymerase KOD-Plus-Neo (Toyobo) (Figure S1). The forward primer 5′-AATCCCAAGCTTCACCATGTACTTCATGAACATCACGTGGGAT-3′ carried a *Hin*dIII restriction site at the 3′-end, whereas the reverse primer 5′-GCCTAGTCTAGATCACTCCCCGATCATGCCGTT-3′ had a stop codon and an *Xba*I restriction site at the 5′-end.

### Cell culture and transfection

Dulbecco’s minimum essential medium (Sigma-Aldrich) supplemented with 10% fetal bovine serum (Sigma-Aldrich) were used for culturing HEK 293 cells. Cells were plated in 3.5-mm dishes 1 day before the transfection and were transfected with 1 μg minigene construct using FuGENE 6 (Roche) by following the standard method. Forty-eight hours after the transfection, we performed reverse transcription polymerase chain reaction (RT-PCR) for the cells.

### RNA extraction and RT-PCR

**Fig. 1:**
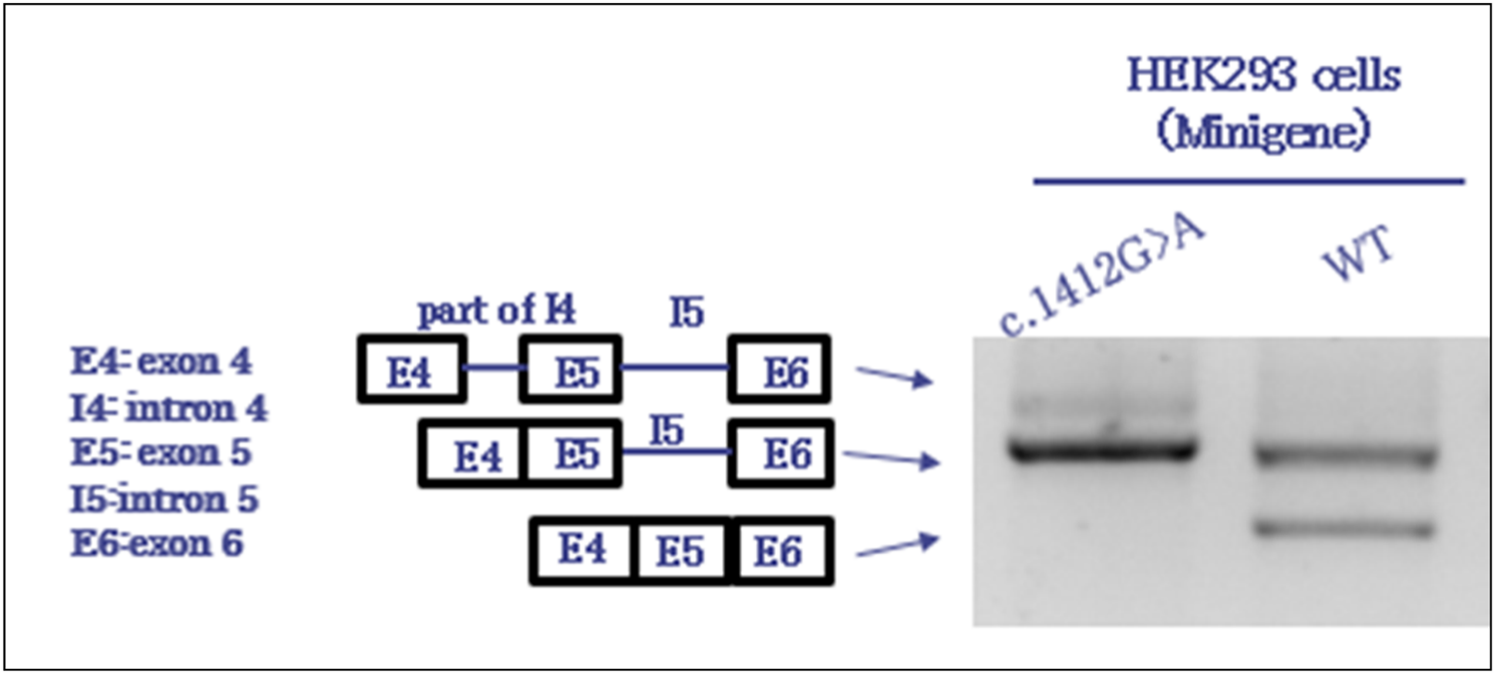
Mutant *GRIN2D* caused intron 5 inclusion

RNeasy Mini Kit (Qiagen) was used to extract total RNA according to the custom methods. Then we synthesized cDNA by using ReverTra Ace reverse transcriptase (Toyobo). GoTaq polymerase (Promega) were used to perform PCR with forward primer designed from exon 4 of *GRIN2D* (5′-CTTCATGAACATCACGTGGG-3′) and reverse primer from exon 6 (5′-GAGTGGCACCTTCCAGGGTC-3′) (Figure S1). Then, we performed agarose gel electrophoresis of PCR products. The correct size bands were excised from the gel by using PCR Cleanup System (Promega) and Wizard^®^ SV Gel and sequenced using Sanger method to confirm the sequence of every band (Fig. 1).

### Follow-up analysis

The statistical power of effective sample size was processed with website program, Genetic Power Calculator (http://zzz.bwh.harvard.edu/gpc/)^39^. With the following parameters: risk rare-allele frequency (A) of 0.01, disease prevalence of 0.01, genotype relative risk (Aa) ≥ 2, genotype relative risk (AA) ≥ 4, marker allele frequency (B) of 0.01, number of cases (*n* = 2259) and controls (*n *= 2040), and type I error rate of 0.05, we computed the result that our sample size have a statistical power of >80%. Only sequences resulting in possible LoF mutations were selected for genotyping. These included one novel frameshift and one novel splice site mutation. Genotyping of the frameshift mutation was performed using a probe oligo that was designed by and ordered from SIGMA-DLP. Genotyping of the splice site mutation was performed with a probe primer (Thermo Fisher Scientific) and Taqman (Applied Biosystems) standard probes. The 384-well microtiter plates were used for analysis, and every plate contained at least one sample carrying confirmed variant and one non-template control. ABI PRISM 7900HT Sequence Detection System (Applied Biosystems) was used for the following analysis with standard method. Then, we compared the mutations with differences allele and genotype frequencies between patients and controls.

## Results

### Results of mutation screening

Our sequence data is available with the accession number DRA004490DNA in the Data Bank of Japan databases (http://www.ddbj.nig.ac.jp). Resequencing of the *GRIN1*, *GRIN2A*, *GRIN2C*,* GRIN2D*,* GRIN3A*, and *GRIN3B* coding regions using Ion PGM platform identified 40 rare (minor allele frequency < 1%), nonsynonymous mutations, including 38 missense mutations, one frameshift mutation with 4 bp deletion (P132Fs in *GRIN2C*), and one splice site mutation (c.1412G > A in *GRIN2D*). All mutations were confirmed by using the Sanger method, and all of them were heterozygous. Among them, 10 variants had available DNA for both of their parents. All the 10 variants were then identified to be inherited by Sanger sequencing, and no de novo variants were found. We searched five genetic databases (dbSNP build 143, 1000 Genomes Project, ExAC, HGVD, and iJGVD) and identified eight variants to be novel variants including the two LoF mutations (P132Fs in *GRIN2C* and c.1412G > A in *GRIN2D*). Then, we conducted in silico analysis for the 40 variants and detected 7 missense variants with deleterious prediction in all of the in silico tools we used (SIFT, PolyPhen-2, MUTATIONTASTER). The details and bioinformatics analysis of all 40 mutations are shown in Table 2.

### Results of splice site mutation

The splice site mutation c.1412G > A-*GRIN2D* revealed a novel G-to-A transition (NM_000836.2: c.1412G > A) at the last nucleotide of exon 5. The predicted results due to splicing according to in silico tools were as follows: SD-Score^36^ predicted c.1412G > A-*GRIN2D* to be aberrant, the human splicing finder^40^ predicted the variant to most probably affect the splicing, and maximum entropy modeling (MaxEntScan)^38^ assigned the mutation a score of −1.87 points, whereas the normal sequence had a score of 5.49 points (Table 3). Then, we performed a functional splicing reporter minigene assay. In Fig. 1, the image shows the resequencing results of the RT-PCR products. Highest bind from the mutant confirmed to containing exon 4 (114 bp), part of intron 4 (99 bp), exon 5 (212 bp), intron 5 (267 bp), and exon 6 (169 bp); second bind from mutant and wild type (WT) containing exon 4 (114 bp), exon 5 (212 bp), intron 5 (267 bp), and exon 6 (169 bp); lowest bind from WT containing exon 4 (114 bp), exon 5 (212 bp), and exon 6 (169 bp). Thus, we confirmed an intron 5 retention due to the mutation, which result in meeting premature stop codon (Figure S2). As normal *GRIN2D* were confirmed to have only one isoform (NM_000836.2), we surmise that c.1412G>A-*GRIN2D* will lead to a truncated, incomplete protein product.

### Results of further genotyping

Frameshift mutation P132Fs in *GRIN2C* and splice site mutation c.1412G>A in *GRIN2D* were selected for genotyping in an larger sample set which included 1877 SCZ cases, 382 ASD cases, and 2040 controls for association analysis. The result showed that no mutations were found in the sample set used for genotyping (Table 4). Importantly, both mutations, P132Fs in *GRIN2C* and c.1412G>A in *GRIN2D*, were only present in a single case, not only among 2821 cases and 2040 controls in this study but also never seen in the following databases: dbSNP build 143, 1000 Genomes Project, ExAC, HGVD, and iJGVD. Thus, we considered them as protein-damaging ultra-rare variants.

**Table 4:** Association analysis results of two loss-of-function mutations

Variant	Genotype counts (resequencing)		Genotype counts (association)			*P* value	
	SCZ	ASD	SCZ	ASD	Control	SCZ	ASD
GRIN2C-P132fs	0/1/739	0/0/384	0/0/3576	0/0/384	0/0/4080	–	–
GRIN2D-c.1412G>A	0/1/739	0/0/384	0/0/3576	0/0/384	0/0/4080	–	–

Genotype counts: Homozygote of minor allele/heterozygote/homozygote of major allele

## Discussion

We performed a systematic work of sequencing the coding regions of NMDARs genes in SCZ and ASD, and detected 40 rare, nonsynonymous mutations in this study. Among them, two LoF mutations in two patients suffering from SCZ were identified: one frameshift mutation (P132Fs in *GRIN2C*) and one splice site mutation (with intron retaining) (c.1412G>A in *GRIN2D*). P132Fs in *GRIN2C* was located in the beginning of the sequence (Figure S3), the 4 bp deletion creating a premature stop codon (p.P132FsX192). Another mutation was c.1412G>A in *GRIN2D*, with a G-to-A transition in the last nucleotide of exon 5. Minigene assay confirmed that this mutation resulted in intron 5 retention carrying two stop codons (Figure S2), which may lead to the introduction of premature termination codons, and possibly causing nonfunctional NR2D receptor to be created. Notably, frameshift mutations in some genes, such as *DISC1* ^41^,* NLGN4* ^42^, and *UPF3B* ^43^, were identified in SCZ and/or ASD patients, suggesting that frameshift mutations may increase susceptibility to SCZ and ASD. Other studies associate intron retention with the pathogenesis of SCZ^44^ and other genetic disorders, such as familial partial lipodystrophy type 2 (ref. 45) and limb girdle muscular dystrophy type 1B^46^, which suggested a role for intron retention in the development of genetic disorders. LoF mutations were often assumed to confer greater disease susceptibility than other missense mutations due to disruption of gene or protein function^28^. They were identified as having an increased contribution to SCZ and ASD, especially in functional sets that are closely involved in neurodevelopment^26^. Genome-wide significant association has also been identified between rare LoF mutations and risk for SCZ and other developmental disorders^26,29^. However, it cannot be ignored that LoF variants were detected also in healthy adults^28,47,48^ with surprisingly no deleterious consequences.

Furthermore, we conducted association analysis for P132Fs in *GRIN2C* and c.1412G>A in *GRIN2D*. Both of the two variants are singleton among 2259 cases and 2040 controls, and also have never been noted in the following databases (dbSNP build 143, 1000 Genomes Project, ExAC, HGVD, and iJGVD), which indicated them to be ultra-rare variants. As LoF ultra-rare variants are suggested to be more abundant among cases with psychiatry disorders than controls^49–51^, the two mutations may confer a strong genetic influence on SCZ risk.

In addition to LoF mutations, we also identified 38 missense mutations in SCZ/ASD patients in *GRIN1*,* GRIN2A*,* GRIN2C*,* GRIN2D*, *GRIN3A*, and *GRIN3B*. Among them, six missense mutations were ultra-rare variants. Seven mutations are predicted to be disruptive using all the three in silico tools we used (SIFT, PolyPhen-2, MUTATIONTASTER). Although in silico predictions are questionable, they are still irreplaceable and used widely to predict the impact of missense variants^52,53^. Moreover, two missense mutations in *GRIN2A* (K441N, K590N) and one missense mutation in *GRIN2C* (E514K) are observed to be positioned on glutamate-binding domain, which is highly conserved in primate. Some studies gave the possibility that the ligand-binding regions were more likely to be disruptive than in other domains^54^, which also indicate the importance of ligand-binding domain.

There are several limitations of our study that should not be ignored. First, we only sequenced the coding region of *GRIN1*, *GRIN2A*,* GRIN2C*,* GRIN2D*,* GRIN3A*, and *GRIN3B*. We excluded promoter, intronic, and 5′- and 3′-untranslated regions. Second, in the genotyping analysis, no statistical significance were in our study, it may be because the size of our samples had no sufficient power. Future studies should include a larger sample size to identify a wider range of rare mutations. Third, due to the difficulty of collecting samples of family members, we were only able to do pedigree analysis for a few subjects. Fourth, to avoid ambiguous pathogenicity interpretations, we excluded missense variants from the association analysis. This strict exclusion criterion limits the number of potential confounding factors, which may cause potentially important targets to be missed.

In conclusion, we revealed 40 rare variants including 38 missense mutations, one frameshift mutation, and one splice site mutation by screening the exonic regions of *GRIN1*, *GRIN2A*, *GRIN2C*, *GRIN2D*, *GRIN3A*, and *GRIN3B*. Our result might imply that these mutations increase susceptibility to SCZ and ASD. Furthermore, the observation of the two LoF mutations in *GRIN2C* and *GRIN2D* supports the hypothesis that an increased burden of ultra-rare deleterious mutations could be observed in SCZ, although statistical significance was not obtained in association analysis. In addition, our data also gave more evidences to support the likely role of NMDARs in SCZ and ASD with a neurodevelopmental origin.

## Electronic supplementary material

Supplementary Materials
