# Supplementary material for: Rare loss of function mutations in N-methyl-d-aspartate glutamate receptors and their contributions to schizophrenia susceptibility
Source: Transl Psychiatry. 2018 Jan 10;8:12. doi: 10.1038/s41398-017-0061-y (PMC5802496; doi:10.1038/s41398-017-0061-y)
Supplement: Supplementary file 1 — Supplementary Materials [file 41398_2017_61_MOESM1_ESM.docx]

***Supplementary Materials***

*Rare loss of function mutations in N-methyl-D-aspartate glutamate receptors and their contributions to schizophrenia susceptibility*

*Yanjie Yu^1^, Yingni Lin^2^, Yuto Takasaki^1^, Chenyao Wang^1^, Hiroki Kimura^1^, Jingrui Xing^1,3^, Kanako Ishizuka^1^, Miho Toyama^1^, Itaru Kushima^1,4^, Daisuke Mori^1,5^, Yuko Arioka^1,6^, Yota Uno^1,7^, Tomoko Shiino^1^, Yukako Nakamura^1^, Takashi Okada^1^, Mako Morikawa^1^, Masashi Ikeda^8^, Nakao Iwata^8^, Yuko Okahisa^9^, Manabu Takaki^9^, Shinji Sakamoto^9^, Toshiyuki Someya^10^, Jun Egawa^10^, Masahide Usami^11^, Masaki Kodaira^11^, Akira Yoshimi^12^, Tomoko Oya-Ito^1,13^ , Branko Aleksic^1*^ , Kinji Ohno^2^ and Norio Ozaki^1^.*

*1Department of Psychiatry, Nagoya University Graduate School of Medicine, Nagoya, Aichi, Japan*

*2Division of Neurogenetics, Center for Neurological Diseases and Cancer, Nagoya University Graduate School of Medicine, Nagoya, Japan*

*3Shengjing Hospital of China Medical University, Shenyang, Liaoning, China*

*4Institute for Advanced Research, Nagoya University, Nagoya, Aichi, Japan*

*5Brain and Mind Research Center, Nagoya University, Nagoya, Aichi, Japan*

*6Center for Advanced Medicine and Clinical Research, Nagoya University Hospital, Nagoya, Aichi, Japan*

*7Laboratory for Psychiatric and Molecular Neuroscience, McLean Hospital, Belmont, Massachusetts 02478, USA*

*8Department of Psychiatry, Fujita Health University School of Medicine, Toyoake, Aichi, Japan*

*9Department of Neuropsychiatry, Okayama University Graduate School of Medicine, Dentistry and Pharmaceutical Sciences, Okayama, Japan*

*10Department of Psychiatry, Niigata University Graduate School of Medical and Dental Sciences, Niigata, Japan*

*11Department of Child and Adolescent Psychiatry, Kohnodai Hospital, National Center for Global Health and Medicine*

*12Division of Clinical Sciences and Neuropsychopharmacology, Faculty and Graduate School of Pharmacy, Meijo University, Nagoya, Aichi, Japan*

*13Department of Nutrition, Shubun University, Ichinomiya, Aichi, Japan*

**Corresponding author: branko@med.nagoya-u.ac.jp*

***Contents of Supplementary Materials***

**Figure S1: Mutant *GRIN2D* minigene structure.**

**Figure S2: Two stop codons in *GRIN2D* intron 5.**

**Figure S3: Locations of amino acid changes caused by mutations in the *GRIN2C* and *GRIN2D* genes**

**Figure S1:** Mutant *GRIN2D* minigene structure.


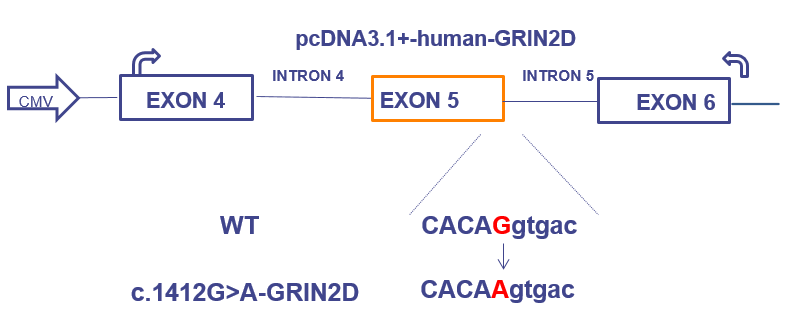


Note: Structure of the pcDNA3.1(+) -human-*GRIN2D* minigene amplifying 5′ end of exon 4 to 3’ end of exon 6. Amplicon size: 1041 bp, containing 114 downstream bp of exon 4, 267bp of intron 4, 212 bp of exon 5, 279bp of intron 5, 169 bp of exon 6.Boxes indicate exons and lines in between boxes indicate introns. The mutation was a G-to-A transition at the last nucleotide in exon 5. Arrows above the exons represent the location of RT-PCR primers. CMV, CMV promoter.

**Figure S2:** Two stop codons in *GRIN2D* intron 5.


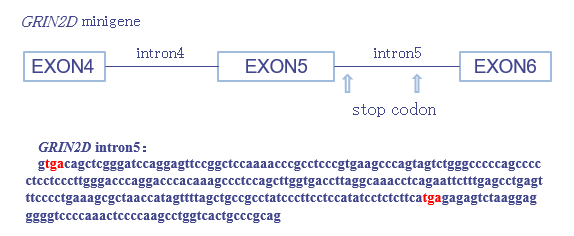


Note: The sequence of *GRIN2D* intron5 is based on human reference sequence NCBI (build 37). The nucleotides in red color are supposed to be stop code.

**Figure S3:** ： Locations of amino acid changes caused by mutations in the *GRIN2C* and *GRIN2D* genes


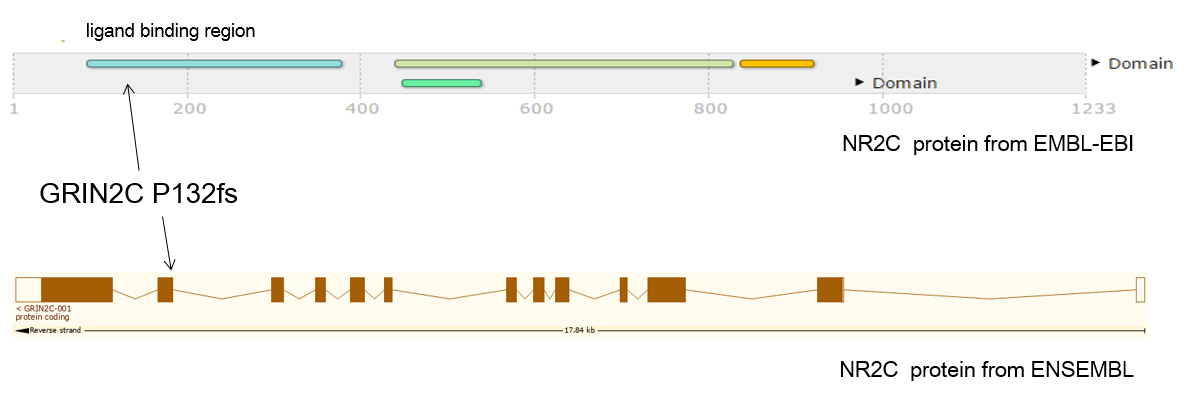


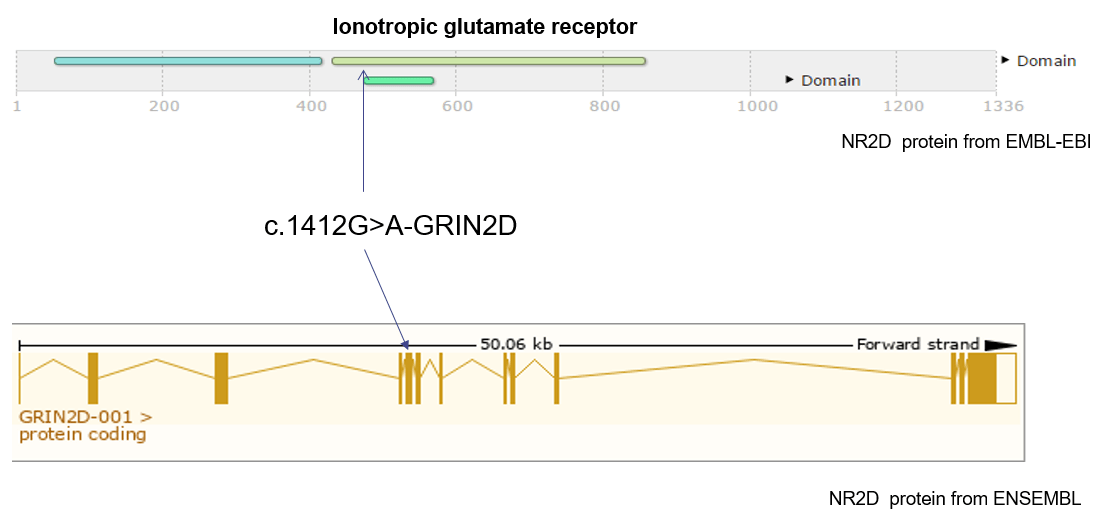


Note: NR2C:N-methyl-d-aspartate receptors subunit 2C; NR2D:N-methyl-d-aspartate receptors subunit 2D.

The structure of protein is based on the EMBL-EBI (European Molecular Biology Laboratory)( https://www.embl.de/) and Ensembl genome database project (https://grch37.ensembl.org/index.html).
